# An Emergency Driving Intervention System Designed for Driver Disability Scenarios Based on Emergency Risk Field

**DOI:** 10.3390/ijerph20032278

**Published:** 2023-01-27

**Authors:** Yuning Wang, Shuocheng Yang, Jinhao Li, Shaobing Xu, Jianqiang Wang

**Affiliations:** 1 School of Vehicle and Mobility, Tsinghua University, Beijing 100084, China; 2 Xingjian College, Tsinghua University, Beijing 100084, China

**Keywords:** driver disability, driving intervention, danger avoidance, risk evaluation, motion planning, automated control

## Abstract

Driver disability has become an increasing factor leading to traffic accidents, especially for commercial vehicle drivers who endure high mental and physical pressure because of long periods of work. Once driver disability occurs, e.g., heart disease or heat stroke, the loss of driving control may lead to serious traffic incidents and public damage. This paper proposes a novel driving intervention system for autonomous danger avoidance under driver disability conditions, including a quantitative risk assessment module named the Emergency Safety Field (ESF) and a motion-planning module. The ESF considers three factors affecting hedging behavior: road boundaries, obstacles, and target position. In the field-based framework, each factor is modeled as an individual risk source generating repulsive or attractive force fields. Individual risk distributions are regionally weighted and merged into one unified emergency safety field denoting the level of danger to the ego vehicle. With risk evaluation, a path–velocity-coupled motion planning module was designed to generate a safe and smooth trajectory to pull the vehicle over. The results of our experiments show that the proposed algorithms have obvious advantages in success rate, efficiency, stability, and safety compared with the traditional method. Validation on multiple simulation and real-world platforms proves the feasibility and adaptivity of the module in traffic scenarios.

## 1. Introduction

Driver disability refers to situations in which drivers lose the ability to drive because of disease or injury, e.g., heart disease, stroke, or heat stroke [1], leading to traffic safety accidents in recent years. Once driver disability occurs, the loss of control of hands and feet inevitably lead to unconventional driving behaviors and probably results in serious traffic accidents [2]. In 2016, 197 truck rollover accidents were caused by sudden disability in Texas, and, unfortunately, all drivers involved passed away due to either disease or the secondary injuries from rollover and collision [3], implying the serious consequences of driver disability. According to the China Statistics of Traffic Accident from 2005 to 2011, fatigue driving, a kind of driver disability, has caused property damage worth about 500 million yuan and 140,000 cases of personal injuries [4]. These facts motivated us to design an automated control system to mitigate the disastrous influence of driver disability.

Most self-driving systems are not designed for taking over when drivers lose their driving ability suddenly. Based on the degree to which the system performs dynamic driving tasks, driving automation is divided into six levels from L0 to L5 [5]. L0 to L2 correspond to driving assistance, requiring drivers to make decisions. L3 to L4 are partial driving automation, in which the driving automation system can perform well in most regular scenarios but requires the driver to take over in specific scenes. L5 is full driving automation, denoting automatic driving wherever and whenever without manual intervention. Although high-level autonomous vehicles where no driver input is needed while driving are highly anticipated, the challenges posed by a high-cost, less robust algorithm pipeline and a lack of general intelligence render it a less mature technology for the near future [6,7,8]. On the other hand, driving assistance technologies, such as ACC (Adaptive Cruise Control) and LKAS (Lane Keeping Assist System), have gained rapid growth and have been equipped on many new vehicle products in recent years. Such vehicles are referred to as autonomous vehicles ranging from L0 to L2 [9,10]. Nevertheless, current driving assistance systems, most of which focuses on regular driving tasks, do not cover emergency accident scenarios [11].

Hence, a novel, enforceable method is required to deal with driver disability. A potential method is to, when driver disability occurs, allow the vehicle fully takes over for the driver to achieve a safe stop automatically, avoiding further human injury and property damage. Referring to a concept in the field of finance, this process is named “hedge” in this paper. The core challenge of the hedge is to plan the motion of the ego vehicle so that it can safely and efficiently navigate amidst static and dynamic traffic participants [12]. In the hedge operation, the driver-disabled vehicle usually must cross several lanes to park in a safe area close to the road, a maneuver during which extra risk is produced compared to regular traffic scenes [13]. For instance, the unexpected lane-changing may lead to wrong predictions and poor decision-making from the drivers of the surrounding vehicles. Therefore, it is critical to quantify and assess the risks comprehensively, which is the core of successful hedging.

This paper aims to solve the emergency hedge issue generated from driver disability scenarios with a newly designed autonomous emergency driving intervention system that includes a feasible risk assessment module named the Emergency Safety Field (ESF) and a corresponding motion-planning algorithm. The paper is organized as follows. Section 2 introduces related work and the contributions of this work. In Section 3, the hedging system’s design is explained. In Section 4, experiments on several virtual and physical platforms are detailed to compare and illustrate the performance of the proposed methods. Section 5 discusses and summarizes this work.

## 2. Literature Review

**Figure 1:**
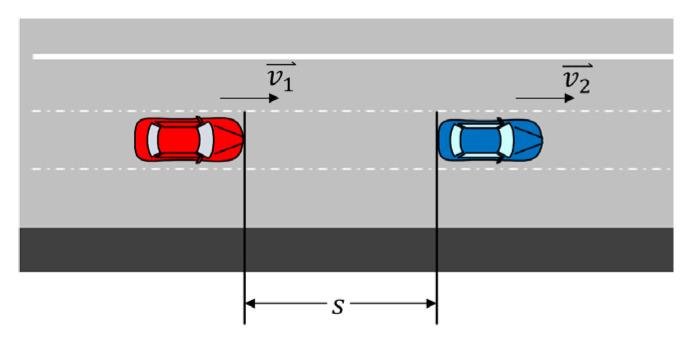
An illustration of TTC.

To park the vehicle in an emergency lane, it is necessary to avoid risky elements on the road. Consequently, it is essential to find a suitable method to describe the traffic risks and plan the motion of the ego vehicle. Some methods have been proposed to assess these traffic risks. These methods can be roughly divided into two types [14,15]: physics-based and statistics-based. Physics-based assessment methods focus on specific scenarios and usually use [14] time-based metrics and kinematics-based metrics to quantify risks. Typical time-based metrics include time-to-collision (TTC) [16], time headway (THW) [17], time to reaction (TTR) [18], and so on, where TTC is defined as the time to a collision, as shown in Figure 1 [19].

The kinematics-based metrics use spatial or spatiotemporal parameters, such as minimum distance [20] and required acceleration for collision avoidance [21], to estimate risk. These methods have the advantage of using a simple and clear physical model with high efficiency, but they only target certain simple scenarios such as car-following or lane-changing. Different from the physics-based methods, the statistics-based methods evaluate risk from historical data [22], including collision probability [23] and machine learning-based metrics. The general idea of collision probability is to use a collision indicator function as the weight of the joint probability density function of the states of the ego vehicle and other objects. The machine learning algorithms depend on learned or handmade features for the classification of risky events. Compared to the former, the learned assessment indicators are more diverse for better comprehensiveness. However, their high computational load and lack of interpretability still pose challenges to decision-making and motion planning.

In addition to the aforementioned methods, another novel assessment method based on artificial potential field (APF) is recognized because of its comprehensiveness and high efficiency: the reported Driving Risk Field (DRF) [24]. The advantages of the artificial potential field method compared with other methods are mainly reflected in local path planning. In the face of unknown or dynamic scenes, vehicles do not need to rely on the prior information of obstacles, as this method has better real-time performance and strong robustness. Ever since its creation, it has been widely applied to risk assessment in intelligent connected vehicles. For example, J. Christian Gerdes et al. applied the APF theory to a lane-keeping system design [25]. Masao Nagai et al. applied APF theory to their pedestrian collision avoidance system at unsignalized intersections [26]. On the basis of previous studies, Wang et al. [24] proposed the concept of DRF to express human–vehicle–road interaction, which analyze the influence of human, vehicle, and road factors on vehicle safety in complex traffic environments. Through continuous improvement, these field theory-based studies have provided real-time and comprehensive assessments in different scenarios. However, fewer studies have focused on emergency scenarios where the safety and efficiency of system operations is of great concern.

Moreover, there exist several reviews that summarize the state of the art of motion planning for autonomous vehicles [27]. On the whole, time-independent path planning has been well-solved using algorithms such as the graph-based planner (e.g., the Dijkstra algorithm, A* family, and state lattices) [28] and the interpolating curve planner (e.g., Bezier, polynomial, and spline) [29,30]. When a field-based approach will be used for risk assessment, APF is undoubtedly more suitable to be selected because it can take full advantage of the risk assessment results and meet the efficiency required in an emergency scenario because of its small computational load. APF is a traditional path planning method in robotics that must be improved if it is to be implemented in vehicles. Chen et al. introduced a velocity vector into the model that can make the robots avoid obstacles while locating and tracking dynamic targets [31]. N. Wahid et al. provided a steering control system based on APF to control the lateral movement of vehicles [32]. However, there is still a lack of a motion planning method based on APF that can cope with emergency scenarios.

This study aimed to design an autonomous hedging system to handle sudden driver disability, focusing on risk assessment and trajectory planning algorithms for emergency parking. The contributions include:

- An emergency safety field (ESF) module designed to assess traffic hazards with the specific aim of autonomous emergency parking in driver disability scenarios.
- A path–velocity-coupled motion planning algorithm based on the ESF module, proposed for pull-over maneuvers in dynamic traffic.
- Verification of the advantages of the proposed method in safety, efficiency, and adaptability with both simulations and real experiments.

## 3. Methodology

**Figure 2:**
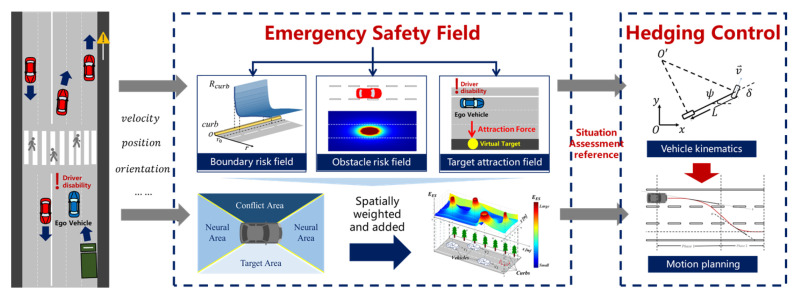
The framework of the autonomous danger avoidance system.

This section introduces the autonomous hedging system design and its two main modules: situation risk assessment based on the emergency safety field (ESF) and the corresponding motion planning algorithm for accident prevention, as demonstrated in Figure 2. The system’s first step is to evaluate the surrounding situation and determine the target destination to guide trajectory planning. Hence, a novel assessment method aimed at emergency hedging events was designed and named the “emergency safety field”. With the input of other obstacles’ physical states involving positions and velocities, three kinds of scenario situation impacts were assessed in the form of field energy. Subsequently, individual fields were spatially weighted according to the features of the emergency hedging task and superimposed to obtain the overall emergency risk field. After deriving a comprehensive and quantitative risk evaluation, a path-velocity coupling motion planning algorithm was designed to realize a safe stop on the roadside.

### 3.1. Emergency Safety Field

#### 3.1.1. Analysis of Emergency Safety

To realize a safe hedge in an emergency scenario, a comprehensive understanding of driving emergency safety is necessary. The elements of a traffic scenario can be classified into three categories, which are the driver, the ego vehicle, and the environment factors. This method was designed under the precondition that the driver has lost the ability to control the vehicle; consequently, human factors were ignored, and we assume that the hardware system of the ego vehicle will support the manipulations ordered via the intervention system. Therefore, the main factors that should be taken into consideration in risk assessment are the environment elements.

Regular elements include moving and static obstacles (vehicles, pedestrians, bicycles, etc.), traffic rules, and road networks consisting of lines and road boundaries. In emergency events, because the safety of the driver and passengers is the first priority, traffic rules and lane lines can be temporarily neglected to realize fast accident avoidance. On the other hand, obstacles and road curbs have great impacts on avoidance because of physical collision possibilities. In addition, in field-based risk assessment models, target directions are crucial elements to ensure validity. Hence, in this work, we considered the road boundaries, obstacles, and target position for pulling over as the three main factors that influence driving emergency safety.

Road boundaries restrict the drivable area and impose hard constraints to the motion planner of the ego vehicle. Obstacles refer to all dynamic traffic elements, such as vehicles, VRUs, mark buckets, etc. During the hedge maneuver, the ego vehicle must avoid these obstacles. In emergency situations, avoidance becomes more difficult than in normal scenarios because of the sudden change of driving objective. When disability scenarios happen, the task of the ego vehicle transitions from regular passing to rapidly pulling over, which leads to more conflicts with other moving objects on the road. The third factor, i.e., the target position, is the most different feature compared with regular driving. In emergency events, the desired destination of the maneuver is a safe area near the roadside instead of driving forward. Therefore, targeted design in situation awareness is required to the propel ego vehicle to the ideal destination.

As previously discussed, the risk field is a good form to represent the comprehensive impact of the aforementioned elements in emergency scenarios. In this section, we propose the Emergency Safety Field (ESF) to model their effects in a unified manner. Correspondingly, three models called boundary risk field, obstacle risk field, and target attraction field were designed from the view of ego vehicle quantitatively. In ESF, the risk of each location to the ego vehicle is expressed as a quantified energy value where a high energy value repels the vehicle away, and vice versa. The details are described below.

#### 3.1.2. Boundary Risk Field

Considering the impact of the curb, the boundary risk field should have two impacts on the ego vehicle: preventing the ego vehicle from driving off the road area and encouraging the ego vehicle to stop in the area close to roadsides (emergency lanes), as this area is safer when accidents happen. Previous models [24] for normal driving do not consider these two requirements; they evaluate risk as inversely proportional to the distance from curbs, repulsing the ego vehicles away from the safe area. Hence, a new boundary risk field is needed.

**Figure 3:**
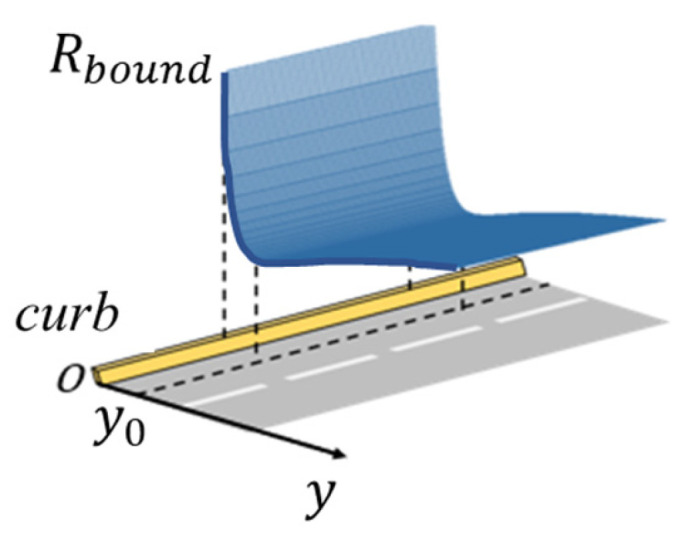
A risk distribution map of boundary risk field.

As shown in Figure 3, in the design, we define y as the offset from the ego vehicle to the boundary, which will change as the ego vehicle changes its position, and $y_{0}$ is the distance from the center line of the emergency lane to the boundary, which is a fixed value. To realize the ideal effects of the boundary risk field, it should generate high risk when $y<y_{0}$ and low risk when $y>y_{0}$. The molecular potential energy model is an appropriate form that provides sharply increasing repulsive forces below the equilibrium point and gravitational forces above the equilibrium point.

Inspired by the molecular interaction effect, we designed the boundary risk field as

(1) $$R_{bound}=-\frac{A}{y^{m}}+\frac{B}{y^{n}}$$

with the following constraints:

(2) $$F\left(y_{0}\right)=\frac{dR_{bound}}{dy}{|}_{y=y_{0}}=0$$

(3)  2<m<n

where $R_{bound}$ denotes the risk road boundary generates, A and B represent the field intensity of different types of road boundaries, and m and n represent the attenuation coefficients. $F\left(y_{0}\right)$ represents the virtual risk force at the center line of emergency lane. Constraint (2) ensures that the risk is minimized at $y_{0}$, and Constraint (3) ensures a sharp risk increase to keep a safe distance to the road boundary.

#### 3.1.3. Obstacle Risk Field

During the emergency hedge process, obstacles, especially those that move at high speeds, are the main source of danger. To avoid physical conflicts with other participants, the obstacle risk field should generate strong virtual repulsive forces to the ego vehicle. The risk moving agents impose on the vehicle should be anisotropic and related to velocity and direction. Zheng et al. [33] proposed an energy model of moving vehicles based on the Doppler effect. Though good results in normal driving scenarios were presented, it fails to satisfy the requirements of an emergency scenario, as it has discontinuity points in the field. The sudden changes that its field intensity suffers may cause sharp jumping at some critical places and result in radical decisions and unsmooth driving control, such as in steering and braking during maneuvers.

**Figure 4:**
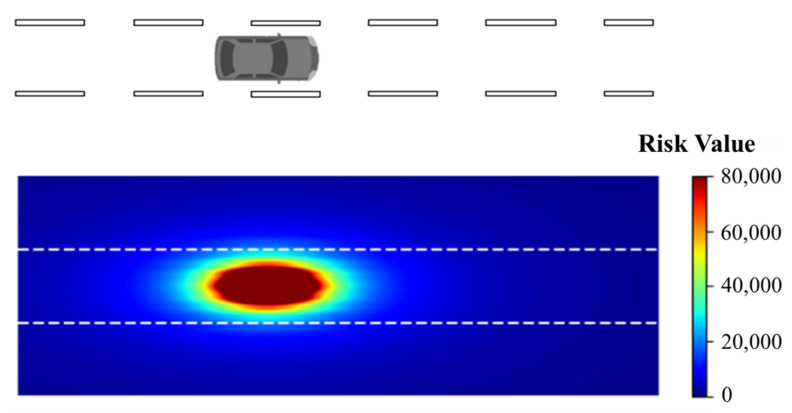
A sample distribution of obstacle risk field.

In this section, we explain how we improved the existing model to adapt to emergency hedging events. Using a Frenet coordinate system denoted by xoy, where x is the axis along the lane and y is the axis perpendicular to the lane, the risk at spot $\left(x_{l},\;y_{l}\right)$ generated by obstacle i locating at $\left(x_{o},\;y_{o}\right)$ is

(4) $$R_{obs_{i}}=R_{0i}\left(\frac{1}{k_{x,0}{}^{2}{\left(x_{o}-x_{l}\right)}^{2}+{\left(y_{o}-y_{l}\right)}^{2}}-\frac{1}{r_{maxi}{}^{2}}\right)$$

where $R_{0i}$ denotes the inherent risk and $r_{\max i}$ represents the cut-off distance. $R_{0i}$ and $r_{\max i}$ are related to the kinds of obstacles present, e.g., pedestrians, sedan cars, trucks, bicycles, etc. In general, $R_{0i}$ is proportional to the mass of the obstacle. For obstacles that are extra dangerous, such as fuel tank trucks, the inherent risk is additionally revised to a larger value. $k_{x,0}$ denotes the direction gradient parameter to describe the anisotropy feature of the obstacle risk, and it is calculated as

(5) $$k_{x,0}=\frac{{\left[v_{\max}-v_{l}\cdot \tan h\left(x_{o}-x_{l}\right)\cdot \tan h\left(v_{lx}-v_{ox}\right)\cdot \tan h\left(v_{ly}-v_{oy}\right)\right]}^{2}}{{\left[v_{\max}+v_{o}\cdot \tan h\left(x_{o}-x_{l}\right)\cdot \tan h\left(v_{lx}-v_{ox}\right)\cdot \tan h\left(v_{ly}-v_{oy}\right)\right]}^{2}}$$

where $v_{\max}$ represents the speed limit of the road, $v_{ox}$ and $v_{oy}$ denote the obstacle’s velocity component along x and y, and $v_{lx}$ and $v_{ly}$ denote the velocity of the object at $\left(x_{l},\;y_{l}\right)$. With the activation of $tanh\left(\cdot \right)$, the continuity of the obstacle risk field is ensured while maintaining the directivity of the field. It is worth noting that for a vehicle running normally, the longitudinal velocity component is much larger than the lateral one; thus, in most practical cases, Equation (5) can be degenerated into Equation (6):

(6) $$k_{x,0}=\frac{{\left[v_{\max}-v_{l}\cdot \tan h\left(x_{o}-x_{l}\right)\cdot \tan h\left(v_{l}-v_{o}\right)\right]}^{2}}{{\left[v_{\max}+v_{o}\cdot \tan h\left(x_{o}-x_{l}\right)\cdot \tan h\left(v_{l}-v_{o}\right)\right]}^{2}}$$

where $v_{l}$ and $v_{o}$ denote the velocity of the obstacle and the object’s location, respectively. Figure 4 shows a sample of the obstacle risk field of a moving vehicle given $R_{0i}=5000$, $r_{\max i}=20\;m$, and a velocity of 40km/h.

#### 3.1.4. Target Attraction Field

The most different feature between normal driving and emergency hedging is the setting of destination. During normal driving, the objective is to go forward with a lower cost, including time, safety risk, fuel consumption, vibration, etc. However, in emergency hedge events, the main task is to stop by the roadside as soon as possible. The local target changes from a forward point to a parallel line close to the curb. Previous methods of target destination modelling [34] did not consider such demands, and a new design is needed.

In this work, we designed the assessment field of the target destination to reflect demand based on the emergency scenario’s essence and on objective analysis. The field model has three characteristics. First, based on the features of field-based risk assessment models different from the repulsive forces from boundaries and obstacles, the driving target should impose an attractive force on the ego vehicle. Attractive forces cause risk energy to decrease so it can reach the destination via gradient descent motion planning. Second, the position of the target point can change along a trajectory parallel to the roadside according to traffic conditions. In an emergency situation, it is dangerous for a vehicle to suddenly stop; instead, it must drive forward slowly while trying to find a window to cut into the safe lane, which makes the ideal stop point also move forward. Third, time efficiency is also a concern in an emergency. The intensity of target field should thus be well-designed to generate sufficient attractive force. Great attractive force accelerates the hedge move, saving time for the disabled driver’s rescue.

**Figure 5:**
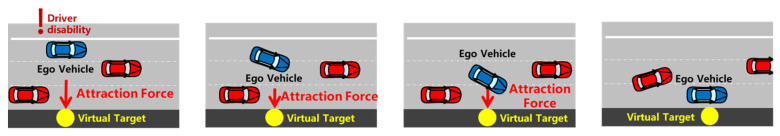
Concept of virtual target attraction.

Based on the above analysis, in this section, a novel target attraction field based on the virtual target attraction concept is explained, as shown in Figure 5. When driver disability happens, a virtual target point at the projection of the accident vehicle is created first, and the virtual target moves along with the vehicle. With the continuous attraction force from the virtual target, the ego vehicle is gradually drawn to the roadside until the hedge is finished.

**Figure 6:**
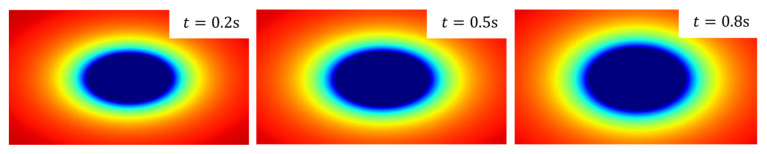
Distribution of target attraction field over time.

To realize the designed effect, a target attraction field for the virtual target is a must. Assuming the location of the accident (ego) vehicle is $\left(x_{a},\;y_{a}\right)$, then the site of its projection on the emergency lane should be $\left(x_{a},\;y_{0}\right)$, where $y_{0}$ has the same definition in boundary risk field. Hence the target attraction field intensity $A_{target}$ on the spot $\left(x_{l},\;y_{l}\right)$ is designed as

(7) $$A_{target}=\frac{A_{0}t^{2}}{k_{a}{}^{2}{\left(x_{a}-x_{l}\right)}^{2}+{\left(y_{0}-y_{l}\right)}^{2}}$$

where $A_{0}$ is a constant related to the type of the accident vehicle, t is the time duration since the accident occurred, and $k_{a}$ denotes the direction gradient parameter as calculated with Equation (8):

(8) $$k_{a}=\frac{v_{\max}{}^{2}}{{\left[v_{\max}+v_{a}\cdot \tan h\left(x_{a}-x_{l}\right)\cdot \tan h\left(v_{a}\right)\right]}^{2}}$$

where $v_{max}$ is the velocity limit of the road and $v_{a}$ is the velocity of the accident vehicle. An example of the target attraction field is shown in Figure 6. As the system running time increases, the intensity of the attraction becomes larger, encouraging more aggressive behaviors for emergency parking when necessary.

#### 3.1.5. Field Superposition

Based on risk modeling related to road boundaries, obstacles, and the virtual target destination, the assessment of individual factors was quantified with a unified form. The next step is to generate an overall assessment of the emergency scenario. In previous works [24,33], the risk values are simply added. With equal superposition, the risk is isotropous for the ego vehicle, which could be less reasonable in an emergency.

**Figure 7:**
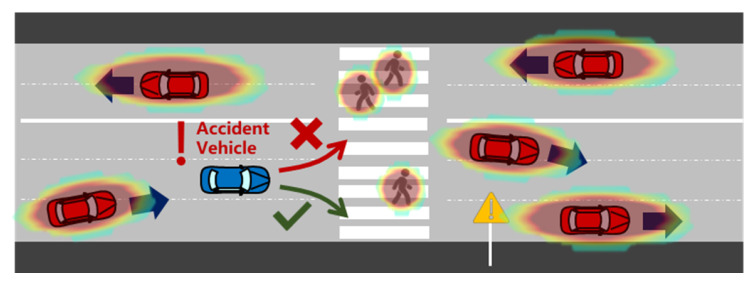
Regional differences for accident vehicles in emergency scenarios.

As shown in Figure 7, complicated driving scenes consist of moving vehicles and pedestrians. In this situation, according to the risk assessment of an individual, it is possible for the accident vehicle to go left or right because the risk there is low. However, for an accident vehicle whose main objective is to stop near the curb, turning left towards the center of the road would be detrimental because it would take a longer time to reach the emergency lane. In an emergency hedge event, the surrounding risk is anisotropic [35]. The side closer to the curb should have relatively less risk.

**Figure 8:**
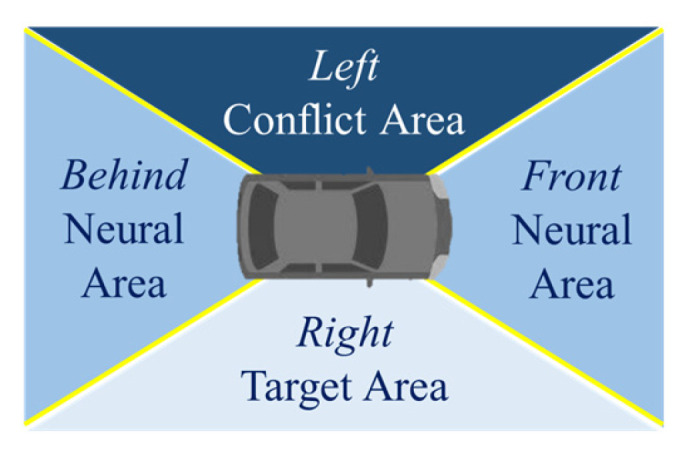
Risk distribution around the ego vehicle.

According to traffic rules and features of traffic flow, we split the space around the ego car into 4 areas, as shown in Figure 8, including the left and right conflict areas and the front and behind neural areas. We assigned higher and lower weights to the left and right areas, considering the features of both risk and targets.

Consequently, the field weight index $w_{ij}$ at $\left(x_{l},\;y_{l}\right)$ is set to

(9) $$w_{l}=\left\{\begin{array}{l} w_{high},\;\;\;\;\;\;\;\;in\;conflict\;area\\ 1,\;\;\;\;\;\;\;\;\;\;\;\;\;\;\;in\;neural\;area\\ w_{low},\;\;\;\;\;\;\;\;\;in\;target\;area\;\;\; \end{array}\right.$$

where $w_{high}$ and $w_{low}$ mean the weights in the conflict and target area, respectively, and they are set to 1.2 and 0.8, respectively, in the following experiments. To ensure the continuity of risk fields, linear interpolation was used in the spaces between them. The final weighted assessment in driver disability scenarios is

(10) $$E_{S}^{l}=w_{ij}\left(R_{bound}^{l}+\underset{O}{\sum }R_{obs_{i}}^{l}-A_{target}^{l}\right)$$

where $E_{S}^{l}$ denotes the overall risk assessment, O denotes the set of all involved obstacles, R means the source generating repulsive effect, and A represents the attractive effect, also called negative risk.

**Figure 9:**
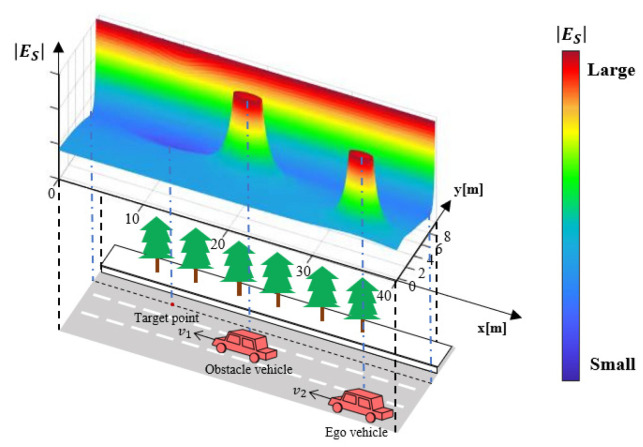
Overall effect of emergency safety field.

An instance of the risk field is shown in Figure 9. The field energy is continuous and distributed on all spaces within the region of interest. Using the regional weighting design introduced above, the risk energy on the dangerous side is increased, whereas that of the safe side is decreased. Based on gradient descending planning, the accident vehicle can follow the route with the lowest risk energy to drive to the emergency lane safe and sound. The next section introduces detailed algorithms to generate real-time motion control commands for the ego vehicle.

Starting with the demands and features of driver disability scenarios, in ESF, we designed three subfield modules and applied the spatial weighting method to realize risk assessment in emergency events.

### 3.2. Motion Planning

Given the risk assessment in emergency scenarios, a reasonable trajectory is required to achieve a safe emergency stop. In this phase of research, we temporarily ignored the effects of suspension because the vehicle is running at low or medium speed in most scenarios during the hedge process and because the effect on motion planning is slight. Several motion-planning methods have been developed leveraging potential field, for instance, velocity generation based on APF theory [36,37]. However, when applying existing methods to the emergency scenarios this paper focuses on, challenges do exist:

1. Inexecutable generated path: usually, the vehicle’s dynamics are not considered, and the generated path with curvature or acceleration beyond the vehicle’s limits cannot be executed.
2. Unreachable stop state: When the car arrives near the target point, due to periodic gravity, instead of stopping at the target point instantly, the car will periodically move around the target point instead.
3. Unsafe parking pose: Though the vehicle can reach the target point and stop safely, an unsafe parking pose, e.g., with high heading angle error, may lead to secondary accidents.

Ignoring constraints in vehicle dynamics and terminal states will greatly reduce the success rate of hedging. Hence, an improved motion planning method is proposed to fulfill the requirements of this special task.

**Figure 10:**
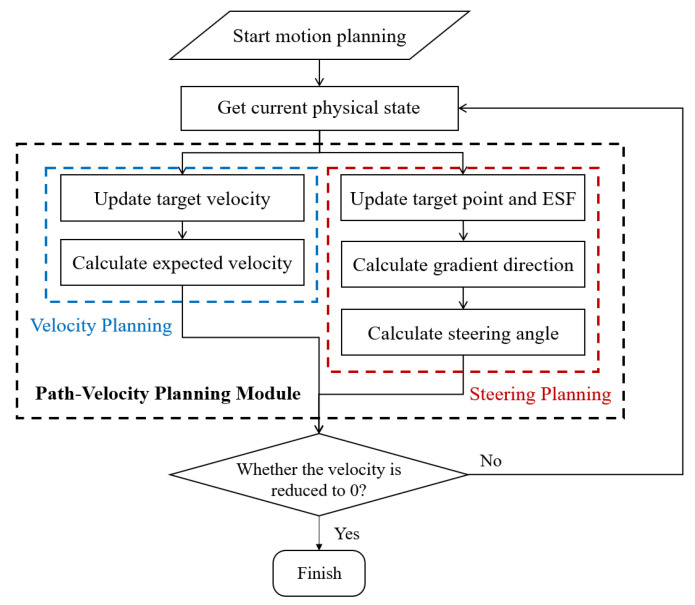
The running process of motion planning.

Figure 10 illustrates the running process of the motion planning, described as follows: When the motion planning starts, first, the current physical states of surrounding vehicles and roads are derived for further assessment. Then, the path–velocity-coupled planning module, consisting of velocity and steering angle planning, begins. As for velocity, first, a function of distance to the curb updates the velocity, and the final expected velocity is calculated based on proportional control. On the other hand, the steering angle requires the result of the ESF. After updating the target point and the value of the ESF, the gradient descending direction is derived. Together with vehicle dynamics and two physical constraints, the final controlled steering angle is output. The velocity and steering angle constitute the final control command. Then, the module judges whether the velocity has reached zero, and if not, the hedge behavior is not over, and so it returns to the first step. Otherwise, the motion planning process ends, and the hedge is finished.

#### 3.2.1. Velocity Planning

First of all, it is necessary to add the expected velocity at each point on the path. Proportional control is used for velocity planning because of its high efficiency and robustness, and a vehicle dynamic model is considered to adjust parameters while constraining the maximum acceleration limit. This is given in Equation (11):

(11) $$\;v_{i+1}=v_{i}+P\left(v_{target}-v_{i}\right)$$

where $v_{i+1}^{\prime }$ is the expected velocity of the ego vehicle at the next time, $v_{i}$ is the actual velocity of the ego vehicle at that time, P is the proportional control parameter used to regulate the speed of vehicle deceleration, and $v_{target}$ is the target velocity, or the speed to which the vehicle must slow down.

In addition, the velocity planning algorithm was modified to associate the target velocity with the vehicle’s position information, enabling the vehicle to decelerate to zero if and only if it reaches the emergency lane. Assume y is the distance from the center line of the emergency lane. Although $v_{target}\left(y\right)$ may be varied, the common boundary conditions, expressed as Equation (12), needs satisfying:

(12) $$v_{target}\left(0\right)=0,\;\;\;\;\;\;\;\frac{dv_{target}\left(y\right)}{dy}>0$$

Hence, to fulfill the requirements of Equation (12), several types of functions were tested. After comparing their performances, the target function was designed as

(13) $$\;v_{target}=v^{*}\cdot \ln\left(y+\frac{y^{*}-y}{y^{*}}\right)$$

where $v^{*}$ is the initial velocity of the accident vehicle when the system is triggered, and $y^{*}$ is the initial distance to the curb. Therefore, combined with Equations (14) and (15), the velocity can be calculated as Equation (14):

(14) $$v_{i+1}=v_{i}+P\left[v^{*}\cdot \ln\left(y+\frac{y^{*}-y}{y^{*}}\right)-v_{i}\right]$$

#### 3.2.2. Steering Planning

As mentioned above, steering planning is closely related to the gradient direction of the risk field. Traditional path-planning algorithms mainly deal with static scenarios, and the target of path planning is a specific point in space; thus, the fixed target points meet the requirements of the model and can effectively play the role of guidance and traction. However, the emergency parking scenario is a dynamic scenario, and it only requires the vehicle to park safely in the emergency lane. Instead of a specific point, the target of the planned path should be a line. If the fixed target point is still used and the target point is set at a fixed place on the emergency lane, the requirements of the model cannot be well-satisfied.

Therefore, this study makes the coordinates of the target point variable in the path-planning algorithm cycle, modifies the function of the target point in the path-planning algorithm, and optimizes the target point from a space point that must be reachable to a gravitational source that provides guidance and traction.

**Figure 11:**
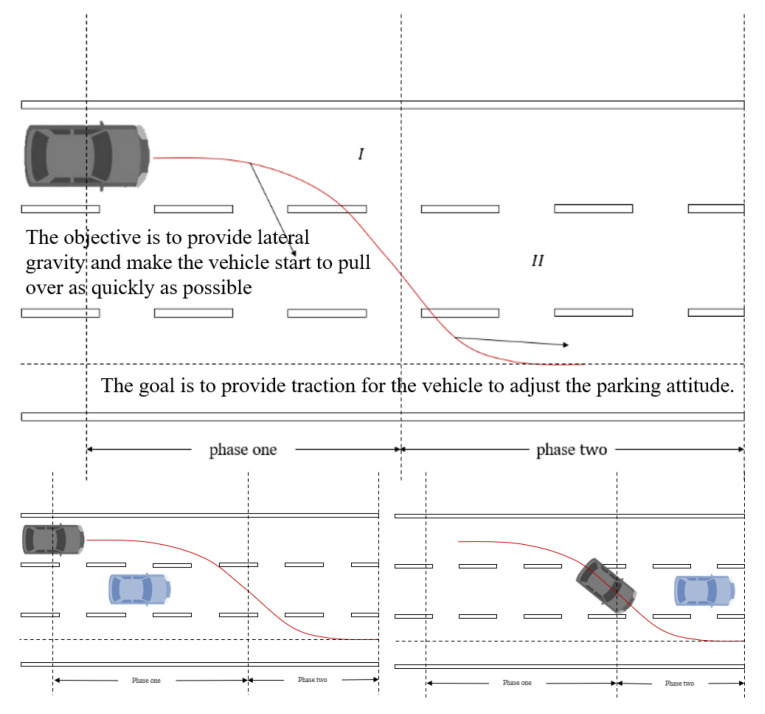
Analysis of variable target thought.

As illustrated in Figure 11, in the first stage, the goal is to provide lateral gravity and make the vehicle start to pull over as quickly as possible. In the second stage, the lateral position of the vehicle has overtaken the second lane. At this time, because the avoidance behavior of the vehicle in the second lane has been completed, the goal is to provide traction for the vehicle to adjust its parking attitude. The following formula is used to fit the position of the target point:

(15) $$x=\left\{\begin{array}{ll} x_{car} & ,\psi \geq 0\\ x_{car}+(y_{car}-y_{target})cot\psi [1-cos\left(-\frac{\pi }{2\psi_{0}}\psi \right)] & ,\psi >\psi_{0}\\ x_{car}+C & ,\psi \leq \psi_{0} \end{array}\right.$$

where $\left(x_{car},y_{car}\right)$ is the coordinate of the ego vehicle at the next moment, ψ is the yaw angle of the ego vehicle at that moment, which can be accessed, $\psi_{0}$ is the heading angle of the ego vehicle as the system moves from phase 1 to phase 2, which is an adjustable parameter related to the road’s width, and C is a constant so the target point is at a fixed distance in front of the ego vehicle along the road direction.

The location of the target point at that time will be transferred to the *Target Attraction Field* built in Section 2 and update the total emergency safety field. With the input of values of the emergency safety field, the gradient of risk field α could be calculated as the target moving direction at the next moment of ego vehicle with Equations (16) and (17), where $E_{S}^{l}$ is the risk energy of the ESF and $f_{x}$ and $f_{y}$ are the *x* and *y* gradient components of the ESF:

(16) $$\frac{\partial E_{S}^{l}}{\partial l_{\theta }}=f_{x}\cos\left(\theta \right)+f_{y}\sin\left(\theta \right)$$

(17) $$\;\alpha =\underset{\theta }{\mathrm{argmin}}\frac{\partial E_{S}^{l}}{\partial l_{\theta }}$$

**Figure 12:**
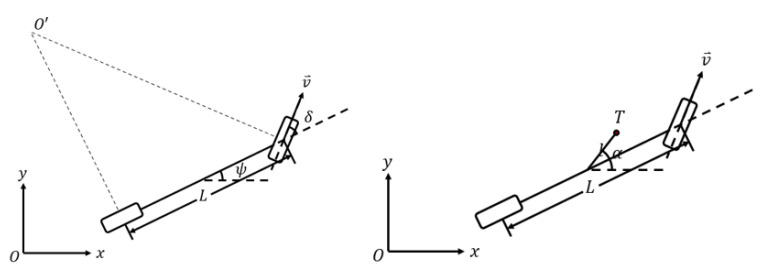
The vehicle geometry and kinematics.

However, as for vehicles, the controlled steering angle varies from the actual moving direction. Therefore, vehicle dynamics must be considered as shown in Figure 12, where ψ is the yaw angle describing the current status of the vehicle center, $\delta_{c}$ is the controlled steering angle, $\vec{v}$ is the velocity of ego vehicle, O′ is the instant center, and L is the wheelbase. After the modeling of dynamics, the relation of the controlled steering angle to the yaw angle is as follows with the same variable definitions as Figure 12:

(18) $$\tan\left(\delta_{c}\right)=2\tan\left(\alpha -\psi \right)$$

Apart from the vehicle’s dynamics, two kinds of constraints are also considered: maximum angular velocity and steering angle, which are defined as $\omega_{0}$ and $\delta_{max}$, respectively. First, the steering velocity constraint is consider. A maximum steering angle variation $\delta_{0}$ exists at each time interval (Δt is time required for each loop), as shown in Equation (19):

(19) $$\;\;\;\;\delta_{0}=\omega_{0}\Delta t$$

and if the variation $\left|\delta_{c}-\delta_{i}\right|$ is below $\delta_{0}$, $\delta_{c}$ remains constant; otherwise, the value of $\delta_{i}\pm \delta_{0}$ with $\delta_{c}$ is assigned to satisfy the maximum steering velocity calculated with Equation (20):

(20) $$\delta_{c}^{\prime }=\left\{\begin{array}{c} \delta_{c},\;\;if\;\left|\delta_{c}-\delta_{i}\right|<\delta_{0}\;\\ \delta_{i}+\delta_{0},\;\;if\;\delta_{c}-\delta_{i}>\delta_{0}\;\\ \delta_{i}-\delta_{0},\;\;if\;\delta_{i}-\delta_{c}>\delta_{0} \end{array}\right.$$

where $\delta_{c}^{\prime }$ is the expected steering angle after one restraint at the next time and $\delta_{i}$ is the actual steering angle of the ego vehicle at that time. Second, the steering angle constraint is considered. If the calculated value $\delta_{c}^{\prime }$ is below $\delta_{max}$, then $\delta_{c}^{\prime }$ remains constant; otherwise, $\pm \delta_{max}$ is assigned to $\delta_{c}^{\prime }$ to satisfy the maximum steering angle as shown in Equation (21), where $\delta_{i+1}$ is the expected steering angle after two restraints at the next time.

(21) $$\delta_{i+1}=\left\{\begin{array}{l} \delta_{max},\;\;\;\;\;\;\;\;if\;\delta_{c}^{\prime }>\delta_{max}\\ \delta_{min},\;\;\;\;\;\;\;\;if\;\delta_{c}^{\prime }>\delta_{max}\\ \delta_{c}^{\prime },\;\;\;\;\;\;\;\;\;\;\;\;\;\;\;else\;\;\;\;\;\;\;\;\;\;\;\;\;\;\; \end{array}\right.$$

After planning the velocity and steering angle at the next moment based on the information at the current moment, the two control quantities constitute a single moment of driving for the ego vehicle. Then, a judgment is made about whether the velocity is 0. If the velocity is 0, then it means that risk aversion has been completed. Otherwise, the cycle proceeds to the next loop of velocity and steering angle planning. The above steps constitute a risk aversion path considering safety, efficiency, and parking pose, accomplishing the goal of pulling over in an emergency scenario.

In summary, based on the characteristics of driver disability scenarios, several specialized modifications are applied in the motion planning module to ensure a safe and sound pull-over maneuver for the ego vehicle.

## 4. Experiments and Results

### 4.1. Experiment Conditions, Metrics, and the Baseline

**Figure 13:**
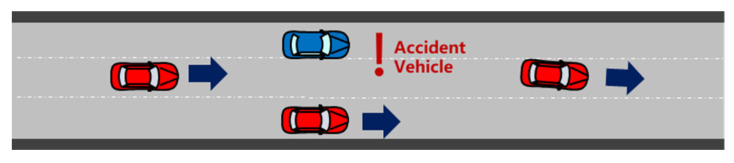
An example experiment scenario.

To test the feasibility and performance of the proposed method, numerous experiments were conducted. Figure 13 shows a sample scenario. A simulation module randomly triggered a driver disability event within 2 s. Six surrounding vehicles were considered and initialized randomly. For each condition, 100 sets of repeated experiments were conducted to ensure credibility.

**Figure 14:**
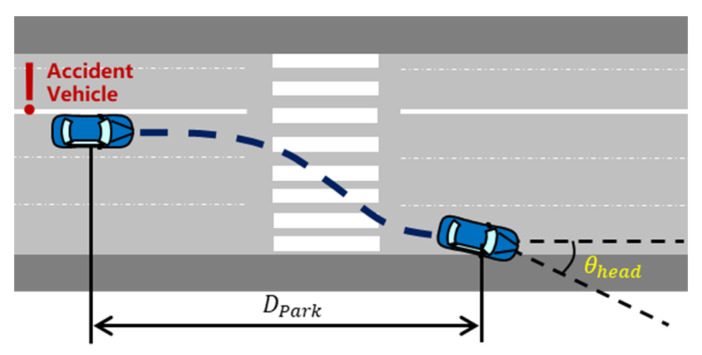
An illustration of parking distance and final heading angle.

The evaluation metrics are important to measure whether the proposed novel framework is better than the existing ones. Two types of indicators are commonly used: direct and composite indexes [38]. The direct indexes have explicit physical meanings, including hedge time $T_{hed}$, parking distance $D_{park}$, the rate of successful hedges $R_{sh}$, and the final heading angle $\theta_{head}$, as shown in Figure 14.

**Figure 15:**
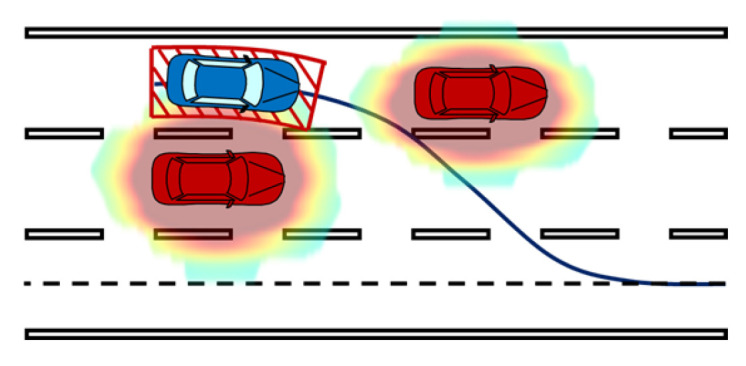
An illustration of parking action.

A composite index combines multiple factors to represent overall performance. In this section, the parking action S is used to reflect the level of danger. Parking action was derived from the Lagrangian action proposed by Zheng et al. [33] and the principle of least action. As illustrated in Figure 15, given a trajectory, the overall risk of this movement is the integral of the risk covered by the red shadow area along the trajectory

(22) $$\;\;\;\;S=\int_{t_{s}}^{t_{f}}\underset{S}{\iint }\left(\sum R_{i}-\sum A_{i}\right)dsdt$$

where $t_{s}$ and $t_{f}$ represent the start and end time, respectively, and S denotes the area covered by the bounding box. The parking action is an integration of the dimensions of space and time during the emergency hedge action. As for the baseline model, we selected the widely used time-to-collision model (TTC) model for risk assessment. The risk of surrounding vehicles is calculated as

(23) $$TTC=\frac{s_{ij}}{\bar{v_{ij}}}$$

where $s_{ij}$ is the relative distance between the ego vehicle and the risk source, and $v_{ij}$ is the relative velocity. TTC denotes the time interval before collision, assuming both vehicles keep constant velocities. If the TTC is below the danger threshold (2s), then the ego vehicle decelerates. If the TTC is above the danger threshold, then the ego vehicle accelerates until it reaches the target velocity.

### 4.2. Experimental Results

**Figure 16:**
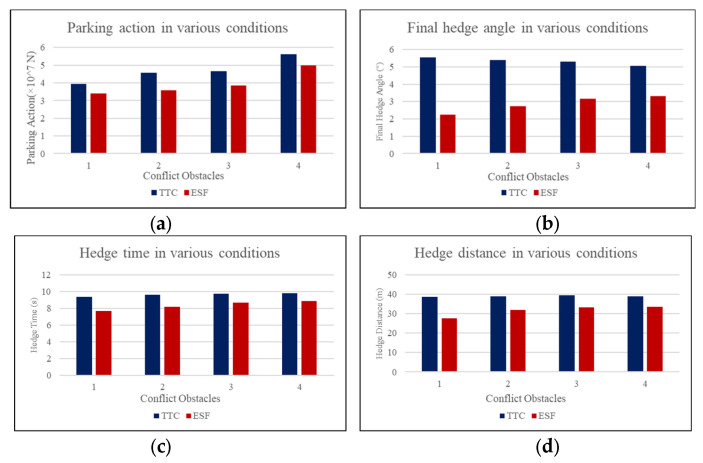
(**a**–**d**) are the comparison results of parking actions, final hedge angles, hedge time, and hedge distances between the TTC and ESF models with various numbers of conflict obstacles.

The experimental results are shown in Table 1. The successful hedge rate of the baseline model was about 5% lower in simple scenarios with only one obstacle and about 20% lower in complicated scenarios with multiple conflict obstacles. When computing the results, for statistics such as the final heading angle, parking distance, and hedge time, all successful cases were counted. As the results of all failed cases were unavailable, they were replaced by the results of the worst successful case to punish the failure reasonably. For example, in 100 sets of experiments with various initial states of four conflict obstacles, the worst successful hedge took 12 s; this value was used for all failed cases. The parking action is a real-time metric not dependent on whether the vehicle stopped. Hence, no amendment was applied on this composite index. When more than four conflict obstacles existed around the ego vehicle, the situation became too crowded to get the vehicle to move; thus, these cases were not compared and are not discussed. Figure 16a to Figure 16d illustrate the comparisons between the baseline and the proposed method.

As shown in Table 1 and Figure 16, the proposed method outperformed the existing ones in all evaluated metrics. First, regarding the successful hedge rate, which is the most important index indicating whether the disabled driver and passengers would survive, the ESF model had an obvious advantage because of its more comprehensive situation awareness. The TTC model did not consider the lateral risk assessment and suffered from crashes with vehicles in side lanes. Due to the features of the field, the ESF model could evaluate all surrounding risks and decelerate and make lane changes when necessary, which led to a higher hedge rate. The higher improvements in more complicated scenarios also indicate better adaptivity.

From Figure 16, we also concluded that from the aspects of time efficiency, parking distances, and heading angles, the proposed method has stable advantages compared with the traditional method. Regarding the heading angle, under the condition of one to four conflict obstacles, the ESF model achieved final pose angles of 59.21%, 49.26%, 40.49%, and 34.46% in descending order. Hedge efficiency is another major strength of ESF, referring to 17.79%, 14.98%, 10.77%, and 9.58% lower parking times and 28.74%, 17.59%, 15.68%, and 14.02% lower parking distances. The parking action shown in Figure 16a shows that the value decreased by 14.18%, 2.84%, 17.6%, and 11.39% with one, two, three, and four conflict obstacles around, respectively.

As a summary, the proposed ESF-based hedge framework performed well on safety, efficiency, and stability, dominating all metrics compared with the commonly used TTC model.

### 4.3. Feasibility Validation

To further demonstrate the feasibility of the proposed methods, we conducted validation experiments on both simulations and physical platforms, i.e., Carla and a mini-scale real-world platform named Sicity.

**Figure 17:**
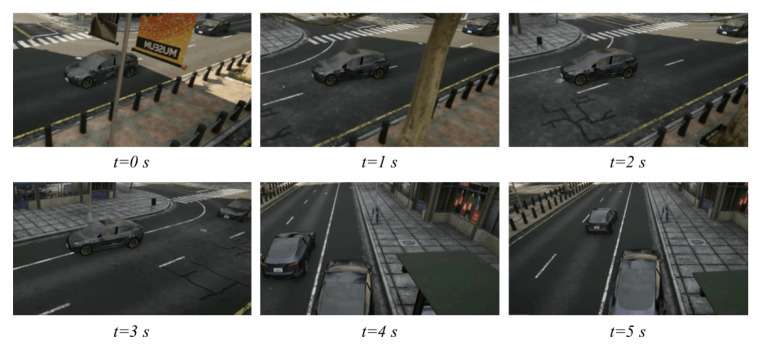
An example of Carla simulations.

Carla is a 3D simulation software developed by Intel, Toyota, and the Computer Vision Center of Barcelona aimed at simulating the real-world traffic behaviors of road participants. In Carla, the city traffic scenario in which all surrounding vehicles are controlled via internal algorithms was selected. The accident vehicle was controlled with external interfaces embedded with the proposed algorithms. An example of the numerical simulations is illustrated in Figure 17. At the beginning, the front vehicle triggered the model to take over. The embedded proposed algorithm started to plan an emergency hedge trajectory with the risk from the rear vehicle controlled by Carla, and it stopped in the emergency lane near the curb successfully.

**Figure 18:**
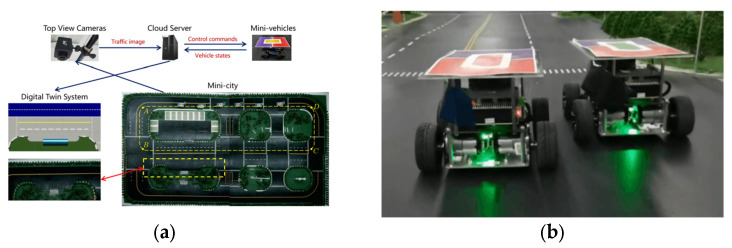
(**a**) An overview of the Sicity platform; (**b**) experimental electric mini-vehicles.

To illustrate the model’s feasibility, we further validated the ESF-based hedge algorithms on a mini-scale real-world platform named Sicity, as shown in Figure 18. Sicity consists of a miniature city to conduct driving behaviors, top-view cameras to capture real-time trajectories, a cloud server for data postprocessing and vehicle control, and mini-vehicles to simulate traffic. The trajectories precepted by the cameras are be sent to the cloud server together with the vehicle states transferred from the V2X communication devices. A virtual digital twin was established for visualization based on the Unity engine. The cloud server also gives control commands to the target mini-vehicles. The electric miniature testing vehicles are shown in Figure 18b, each with a size of 200 mm × 200 mm × 130 mm. A piece of colored cardboard was attached on the top of each vehicle so a vision-based localization module could be utilized to get their real-time positions.

Although the Sicity platform is still different from the actual road, the two have similarities as described below. First, mini-vehicles and actual vehicles both have Ackermann chassis, and their motion at low speeds can be described using the same vehicle kinematics model. Second, the ratio between the Sicity platform and the actual road length is the same as that between the experimental electric mini-vehicles and actual vehicles. In addition, some restrictions were made on the running time interval in the algorithm to simulate the computing power of the vehicle processor in real situations. This method was used to verify the real-time performance of the system. Third, the experimental electric mini-vehicles also use the wireless network to receive and transmit data, which is similar to the architecture of the roadside network connection facilities.

**Figure 19:**
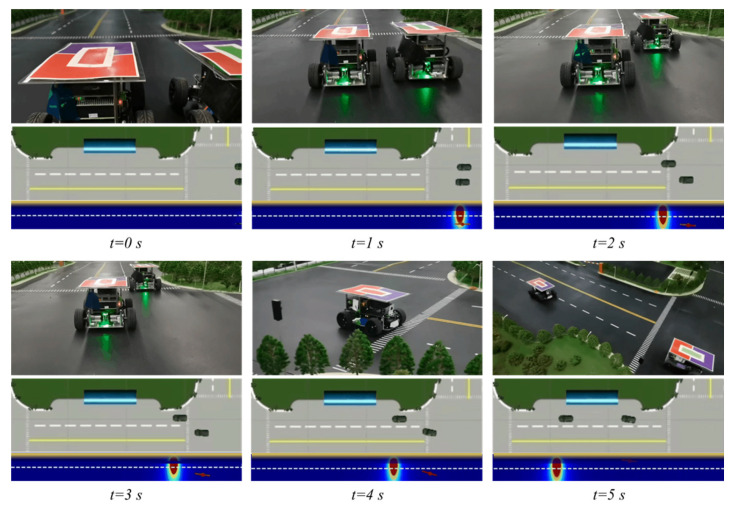
An example case of Sicity platform for real-world experiments.

Figure 19 gives an example case of real-world experiments. The left accident vehicle (AV) was triggered to run the emergency hedge algorithm. To avoid a crash with the right conflict vehicle (CV), it first slowed down and shifted to the safety area near the bus stop at the moment risks were clear on the track. For each frame, the corresponding digital twin is given below. At t=2s, although the CV had passed the EV, the risk it generated was still large and may block a potential hedge; consequently, the EV chose to decelerate and wait. At t=3s, the risk was low enough, and the EV began to change lanes, finally stopping in the temporary emergency lane.

In conclusion, the Carla and Sicity validation experiments proved that the proposed method has good adaption to vehicle dynamics and feasibility. The ESF-based hedge framework is a unified and universal method for automatic hazard avoidance. It does not depend on vehicle models, lane numbers, surrounding obstacles, traffic, or other usual traffic variants, and it can be generalized to other scenarios.

## 5. Discussion

The experiment results show that the proposed novel emergency driving intervention system outperformed the traditional TTC-based on the aspects of safety, efficiency, and stability. Moreover, validations on various platforms proved its adaptability. This section will interpretate the results from the perspective of working mechanisms.

1. **Safety**. As analyzed in Section 3, the main danger during the process of pulling over is that of the surrounding moving obstacles. Previous risk evaluation models such as TTC, THW, etc. only considered one-dimensional danger; thus, the hedge rates around multiple conflict vehicles decreased drastically. In comparison, field-based risk evaluation considered danger caused from all surrounding sources comprehensively, giving a two-dimensional risk reference for the following motion-planning section. Moreover, the novel regional weighting technique also guaranteed that the ego vehicle did not turn to the dangerous side of the road.
2. **Efficiency**. The reason why our proposed method achieved quicker avoidance is rooted in three innovations. First, the improved boundary risk field set the emergency lane as the location of the lowest risk energy so the ego vehicle was more likely to reach it. Second, the application of the virtual target attraction concept accelerated the process of lateral movement. Third, we designed the target attraction field as a time-varying source instead of the traditional static format. Therefore, slow movement was be punished during motion planning.
3. **Stability**. Better stability, which was reflected by a smaller final heading angle, was realized by applying the velocity–steering-decoupled planning framework. By designing the goal function, the ego vehicle adjusted its pose automatically when approached the emergency lane. A smaller final heading angle gives more convenience for later rescue operations.
4. **Adaptability**. Instead of being scenario-driven, the ESF-based method assessed traffic risk in an emergency situation in accordance with human drivers’ cognition mechanisms. Hence, it does not depend on specific traffic conditions and can be generalized to other scenarios.

## 6. Conclusions

This paper explained the design of an emergency driving intervention system to deal with driver disability scenarios. The system consists of a ESF model designed for risk evaluation in emergency scenarios and a path–velocity-coupled motion planner, enabling a safe, efficient, and precise emergency stop. The proposed ESF model has an advantage over the TTC model based on the four indicators of parking action, final hedge angle, hedge time, and hedge distance, which embody a higher hedging success rate, better efficiency, and better safety. Simulated and real-vehicle experiments validated the model’s superiority and feasibility. The hedging system can not only ensure the safety of the driver and passengers, but also minimize the influence on traffic flow and avoid secondary accidents when driver disability occurs. In the future, the proposed hedge algorithm framework can be further generalized to other applications.

## Figures and Tables

**Table 1:** Comparison experiment results of emergency danger avoidance events.

	Conflict Obstacles	Parking Action $\left(\times 10^{7}N\right)$	Final Heading Angle (deg)	Parking Distance (m)	Hedge Time (s)	Hedge Rate
TTC Planning	1	3.95	5.54	38.50	9.33	95.00%
ESF Planning		3.39	2.26	27.54	7.67	100.00%
Improvement		14.18%	59.21%	28.47%	17.79%	5.26%
TTC Planning	2	4.57	5.40	38.77	9.61	84.38%
ESF Planning		3.57	2.74	31.95	8.17	93.75%
Improvement		21.88%	49.26%	17.59%	14.98%	11.10%
TTC Planning	3	4.66	5.31	39.35	9.75	75.78%
ESF Planning		3.84	3.16	33.18	8.70	87.50%
Improvement		17.60%	40.49%	15.68%	10.77%	15.47%
TTC Planning	4	5.62	5.05	38.94	9.81	70.00%
ESF Planning		4.98	3.31	33.48	8.87	83.75%
Improvement		11.39%	34.46%	14.02%	9.58%	19.64%

## Data Availability

Not applicable.
